# Supplementary material for: The matricellular protein CCN5 induces apoptosis in myofibroblasts through SMAD7-mediated inhibition of NFκB
Source: PLoS One. 2022 Aug 2;17(8):e0269735. doi: 10.1371/journal.pone.0269735 (PMC9345366; doi:10.1371/journal.pone.0269735)
Supplement: S1 File — (PDF) [file pone.0269735.s001.pdf]

Supporting information Western Blot raw data

The matricellular protein CCN5 induces apoptosis in myofibroblasts through SMAD7-mediated inhibition of NFκB

Mai Nguyen[1], Min Ah Lee[1], Young-Kook Kim[2], Hyun Kook[3], Dongtak Jeong[4], Seung Pil Jang[5], Tae Hwan Kwak[5], and Woo Jin Park[1,*]

S1 Fig 1. The p53 and NFkB signaling pathways are up-regulated in MyoFBs

S1 Fig 1A.

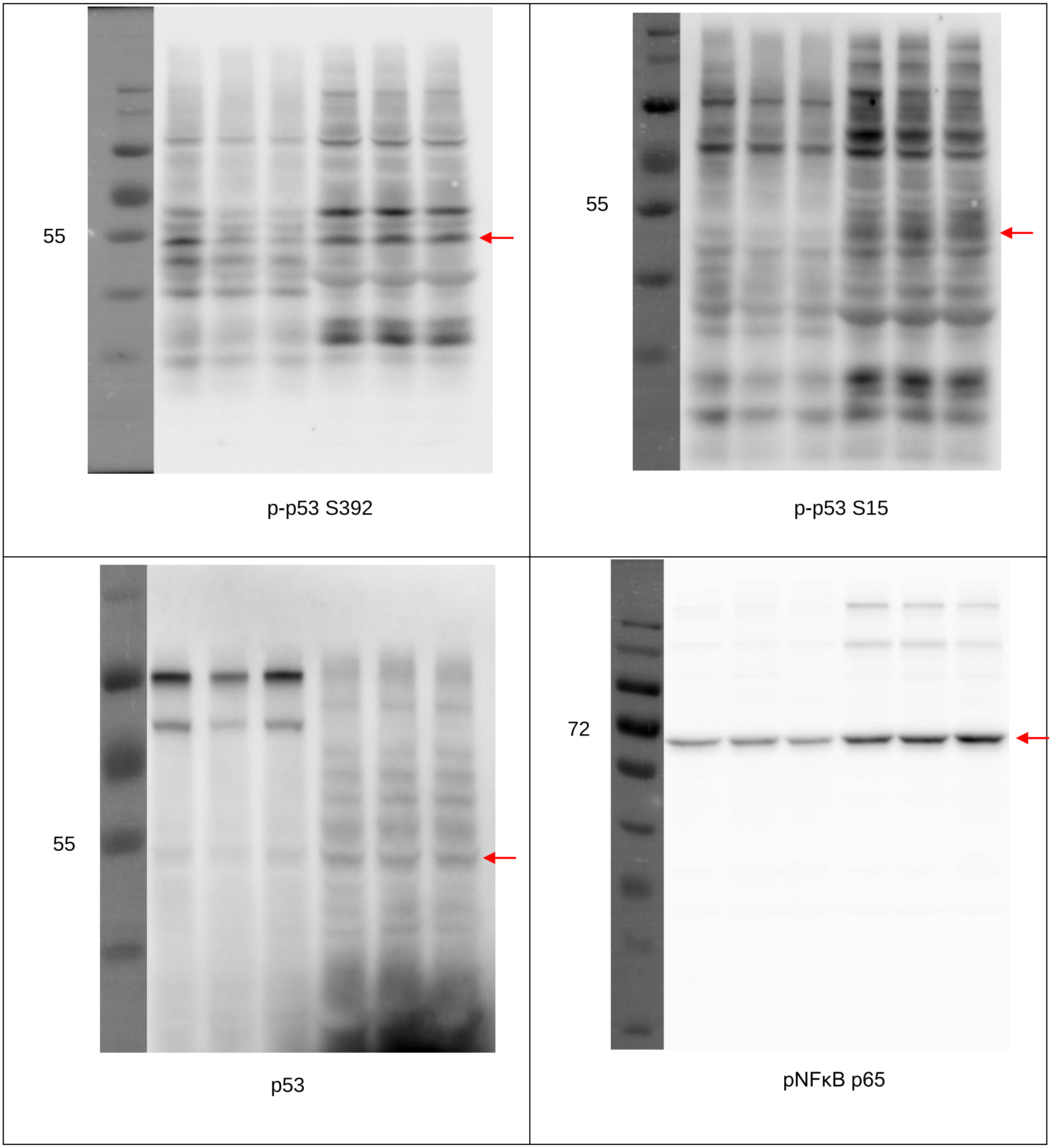

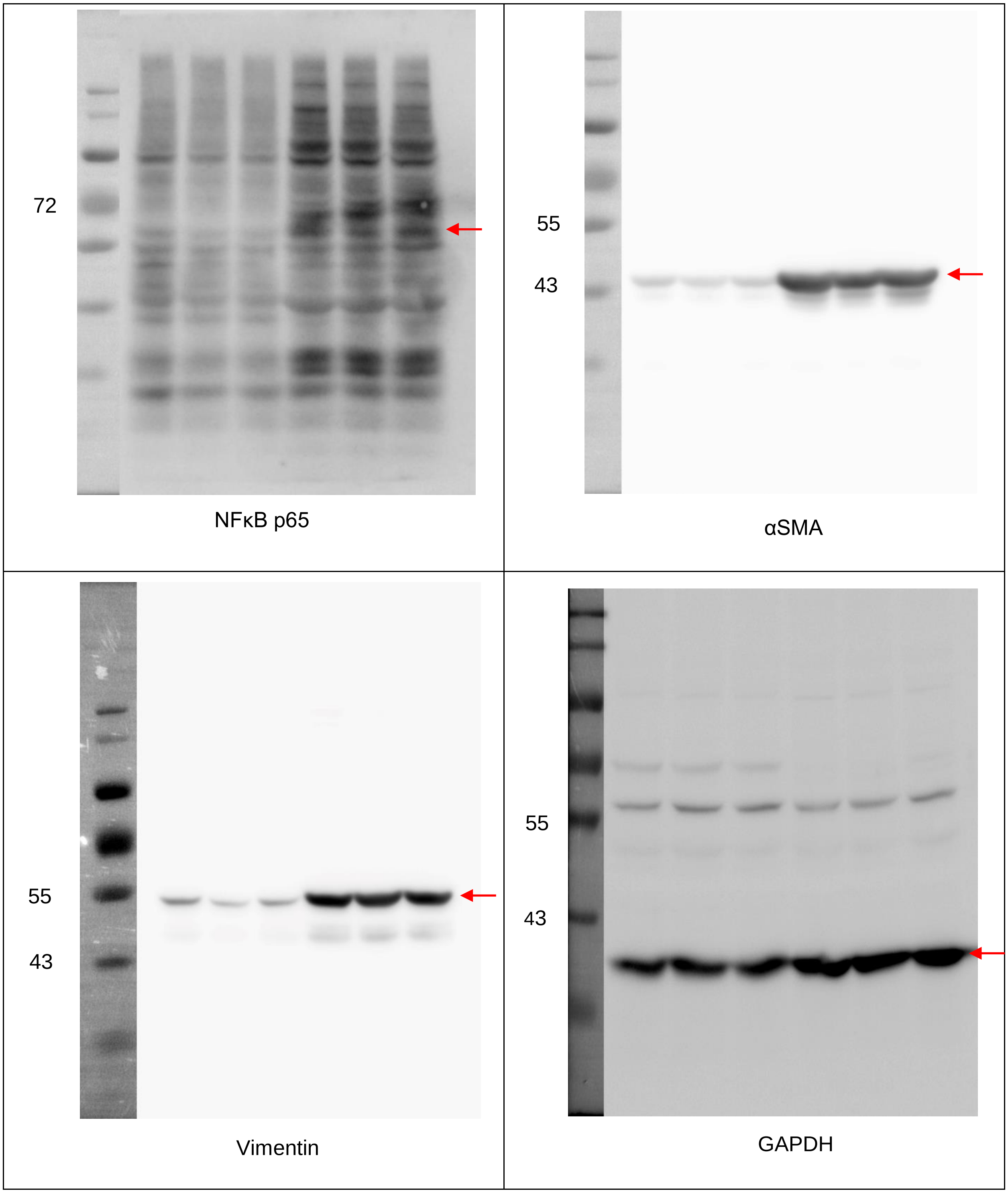
72
NFκB p65
55
43
αSMA
55
43
Vimentin
55
43
GAPDH

S1 Fig 1B.

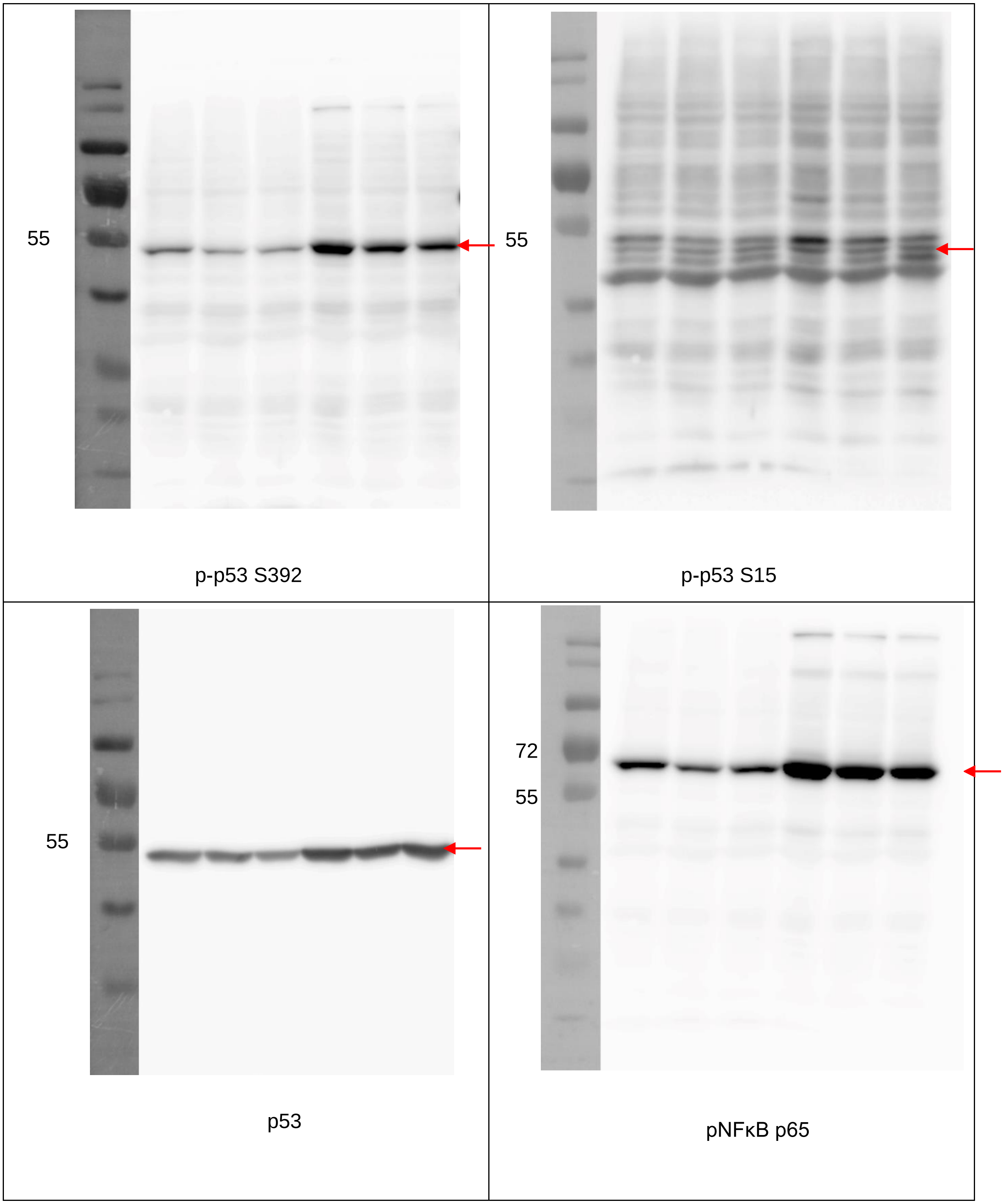

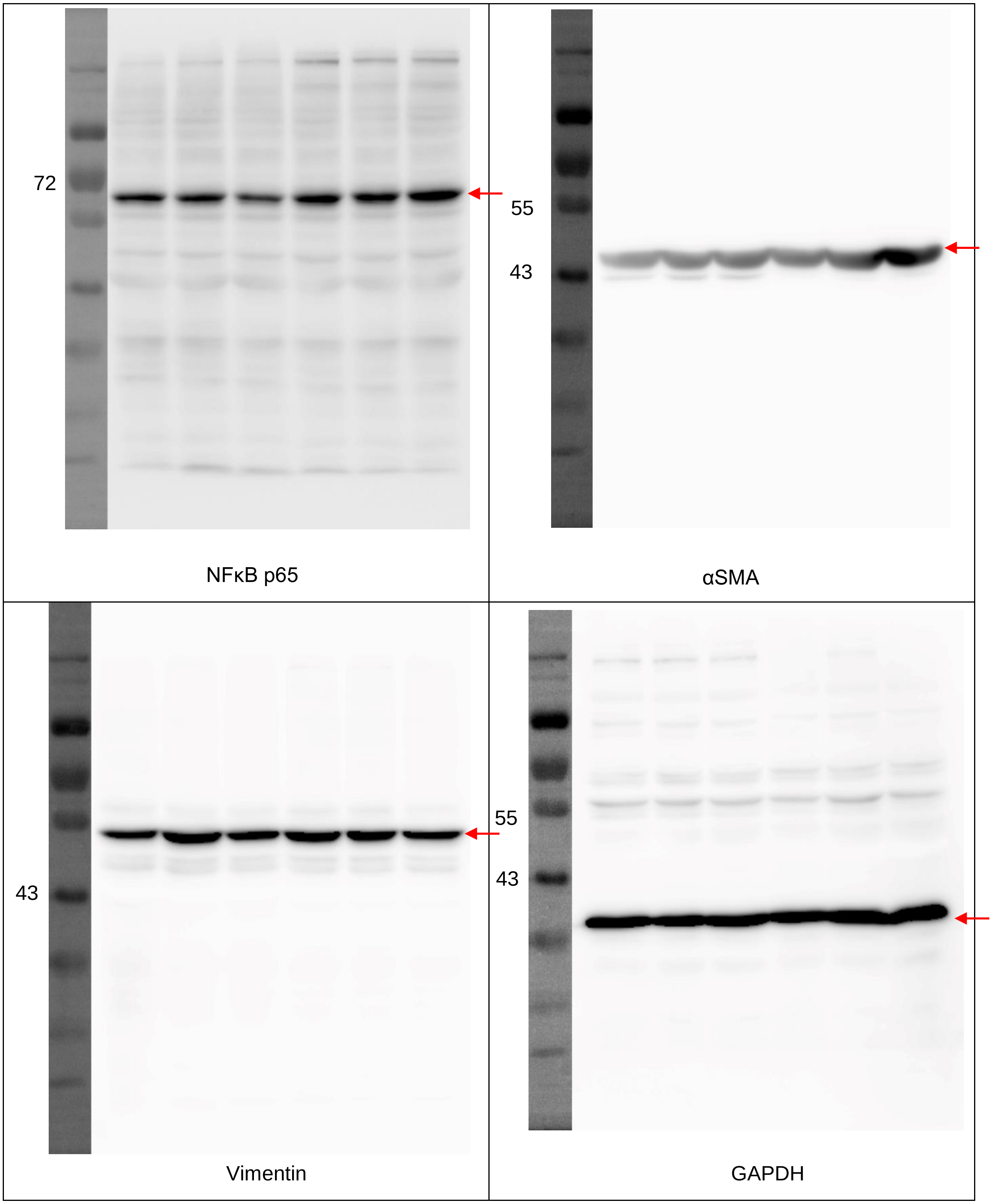
72
NFκB p65
55
43
αSMA
43
Vimentin
55
43
GAPDH

S1 Fig 1C.

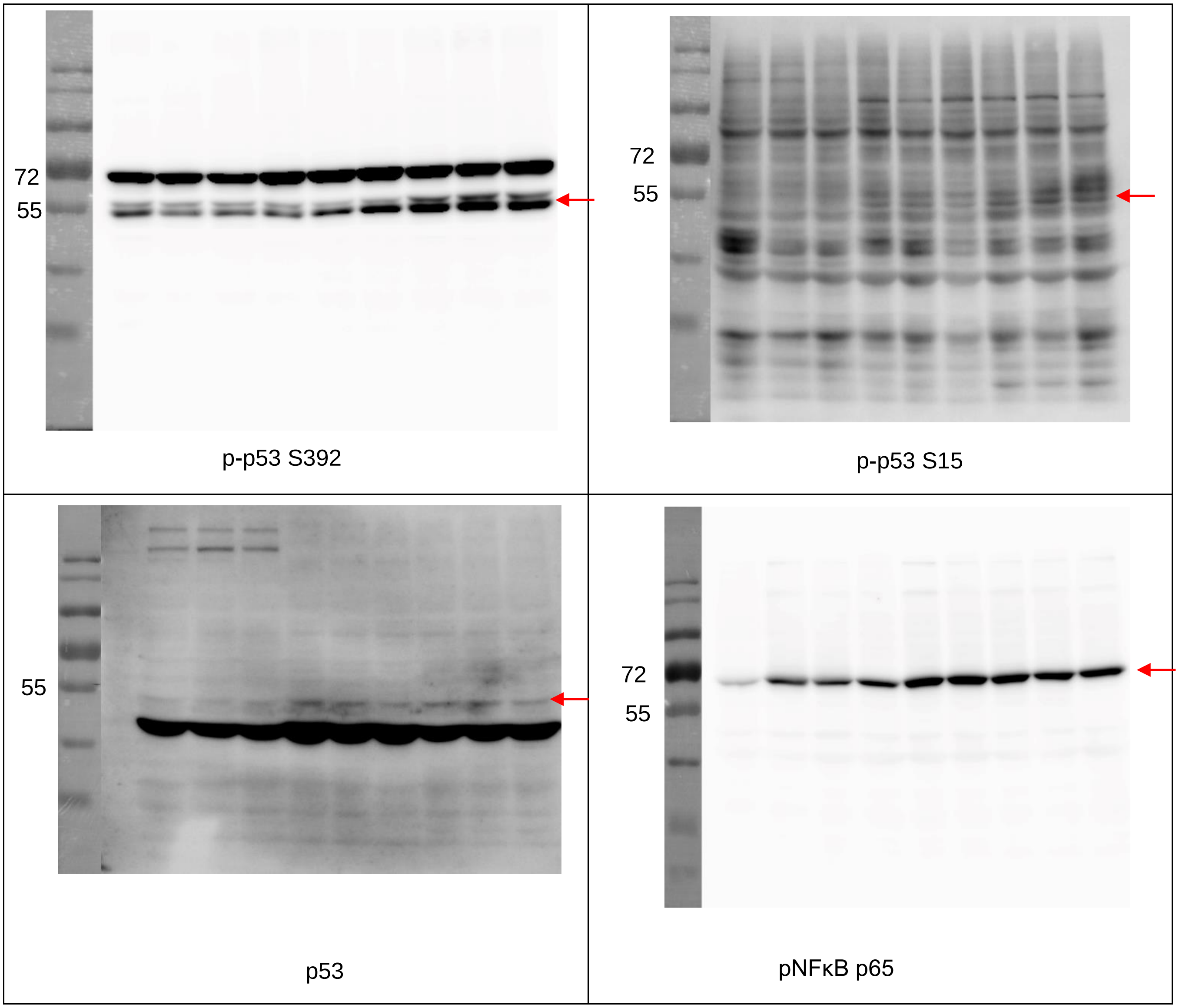

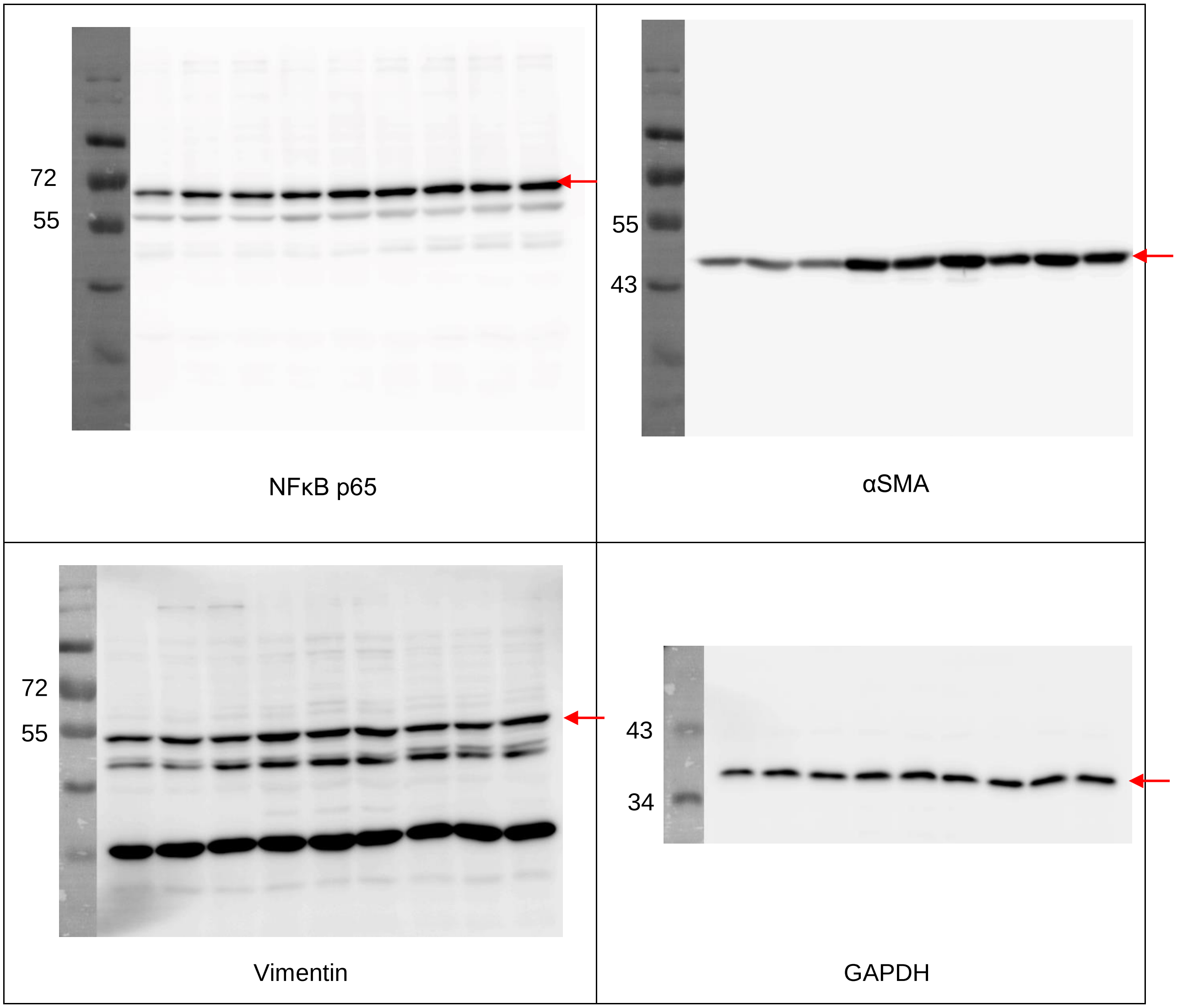
72
55
NFκB p65
55
43
αSMA
72
55
Vimentin
43
34
GAPDH

S2 Fig 2. p53 is involved in CCN5-mediated apoptosis of MyoFBs

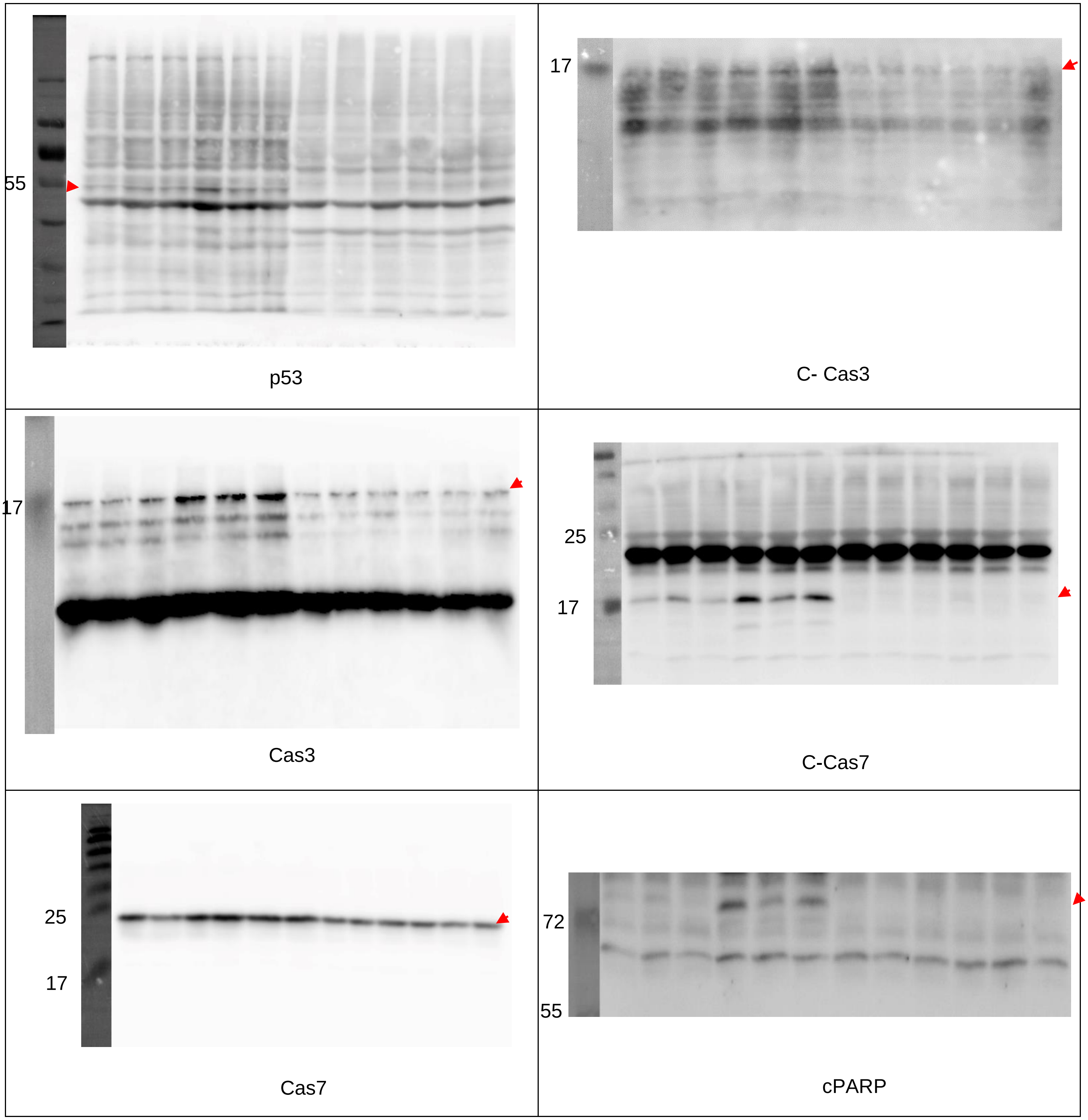

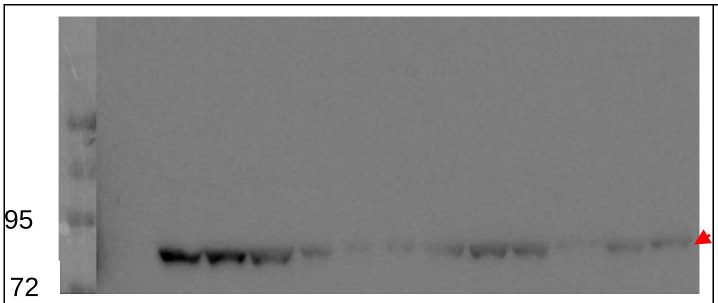

PARP

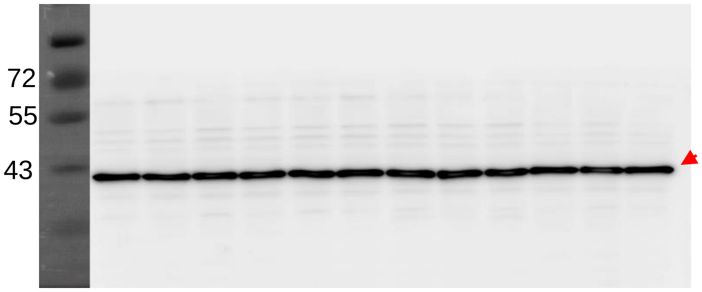

GAPDH

S3 Figure 3. NFkB protects MyoFBs from apoptosis

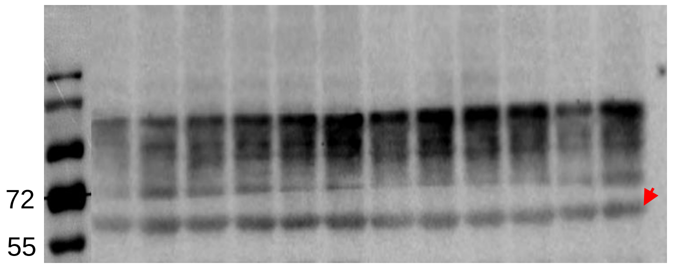

pNFκB p65

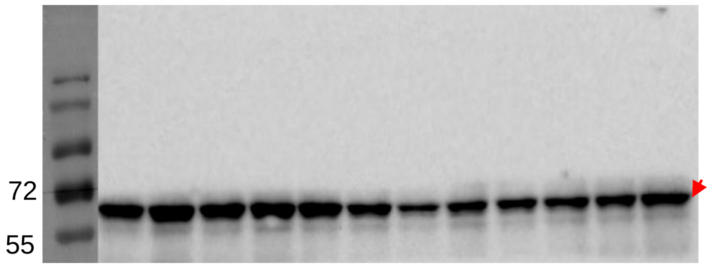

NFκB p65

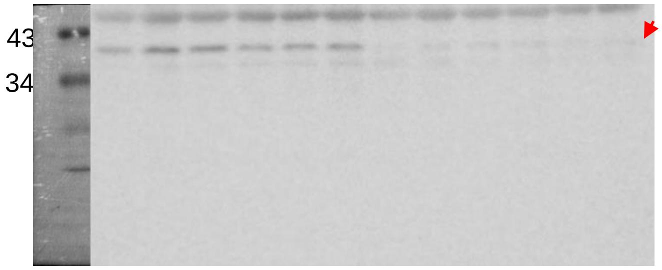

pIκBα

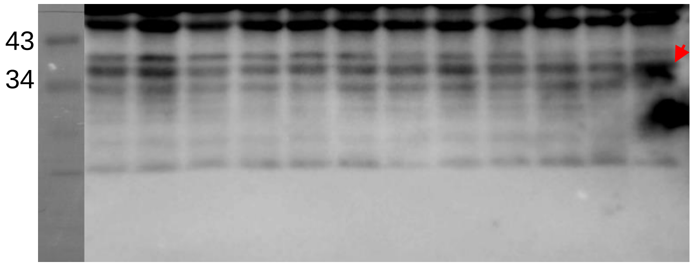

IκBα

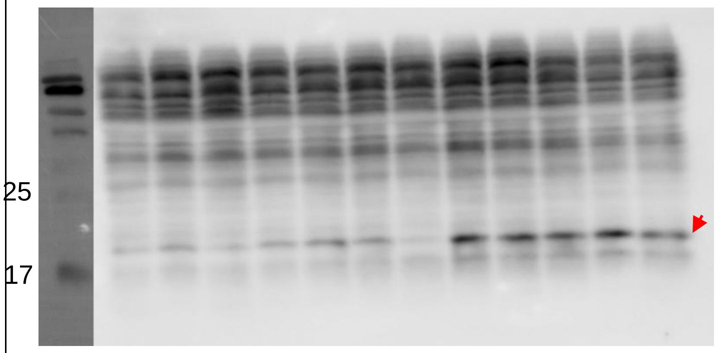

C-Cas3

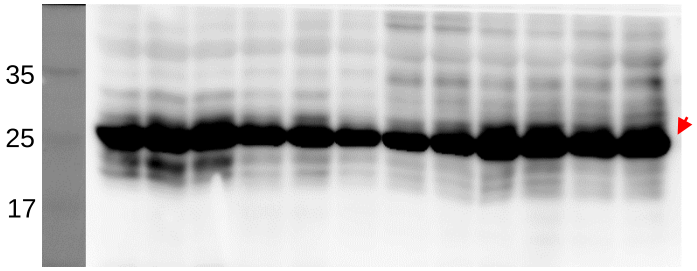

Cas3

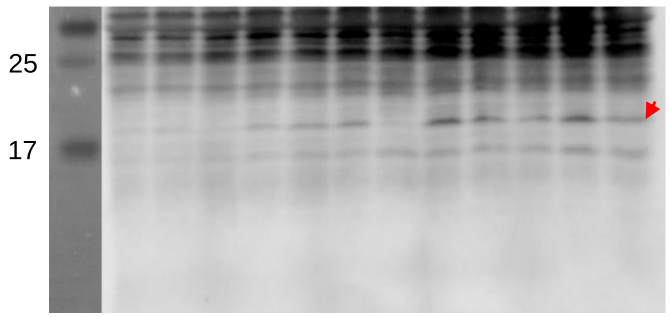

C-Cas7

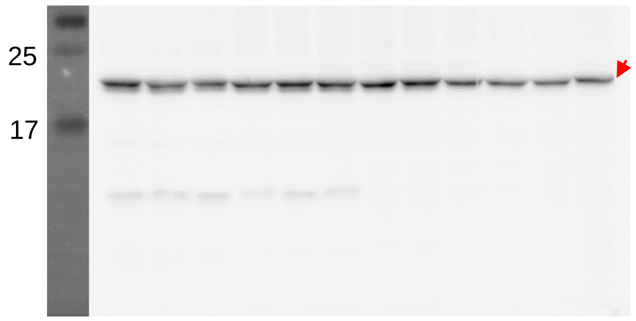

Cas7

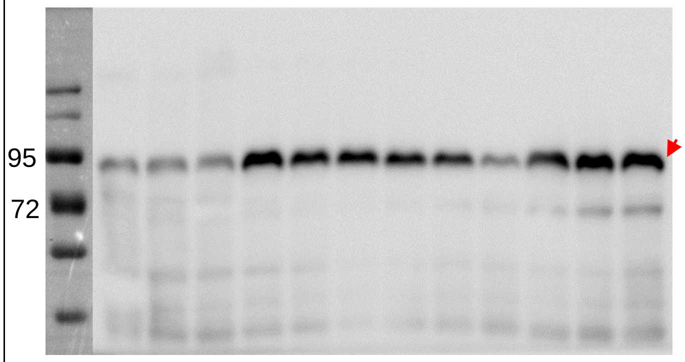

c-PARP

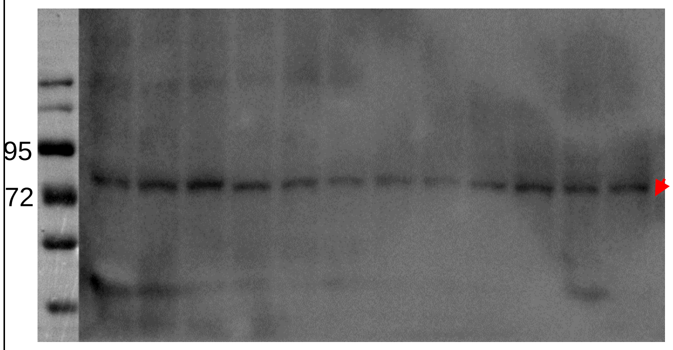

PARP

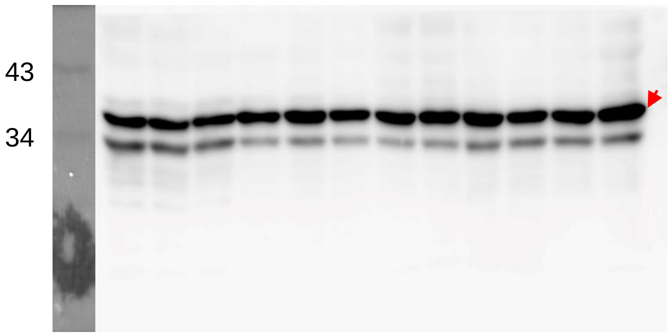

GAPDH

S4 Fig 4. CCN5 inhibits NFkB signaling through elevation of SMAD7 levels

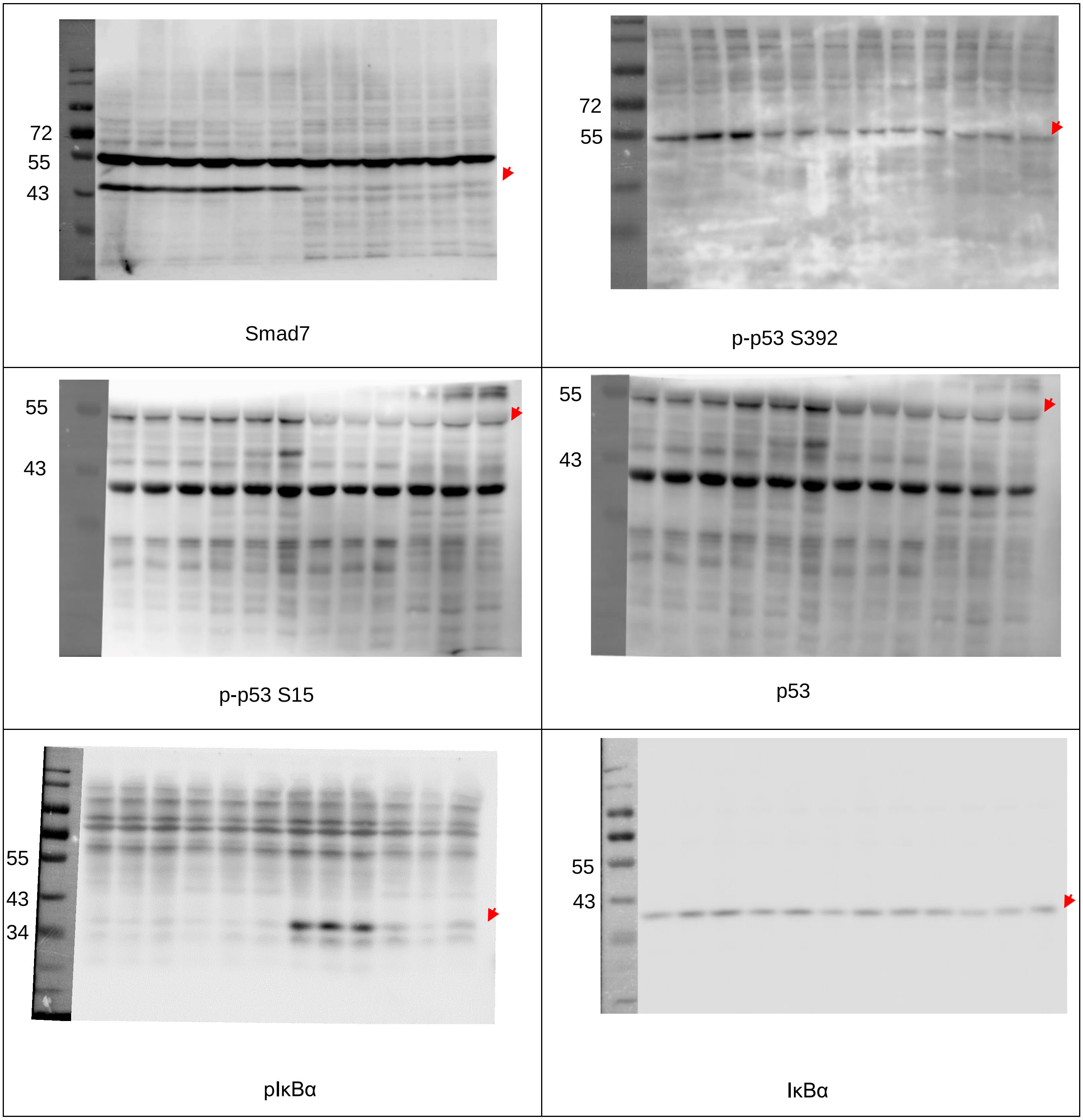

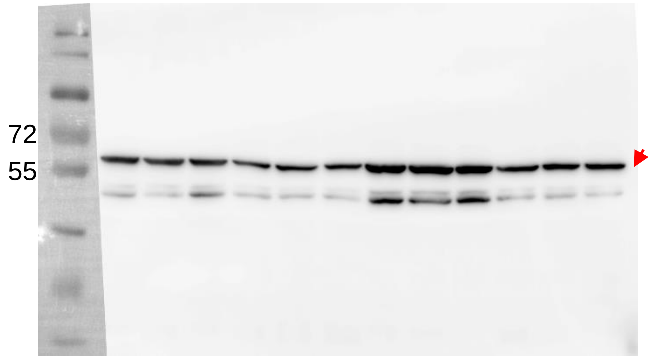

pNFκB p65

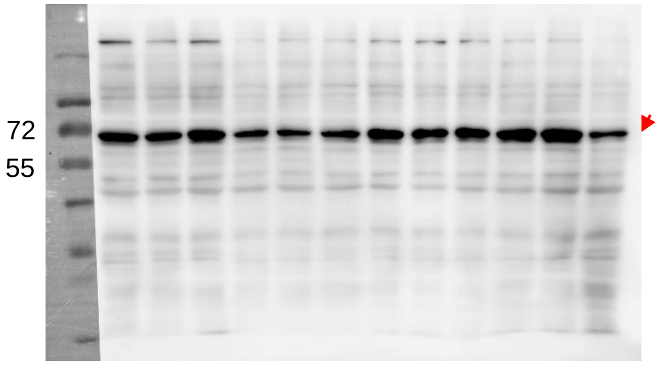

NFκB p65

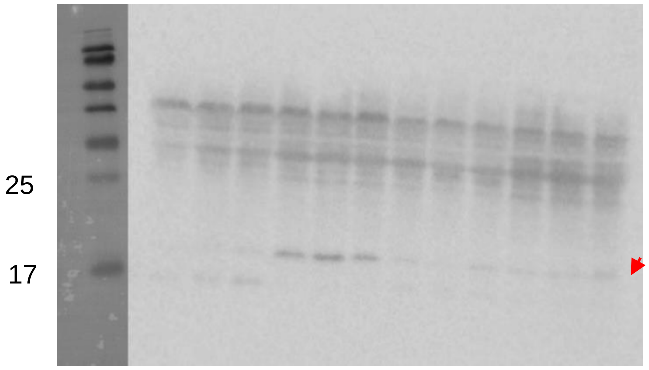

C-Cas3

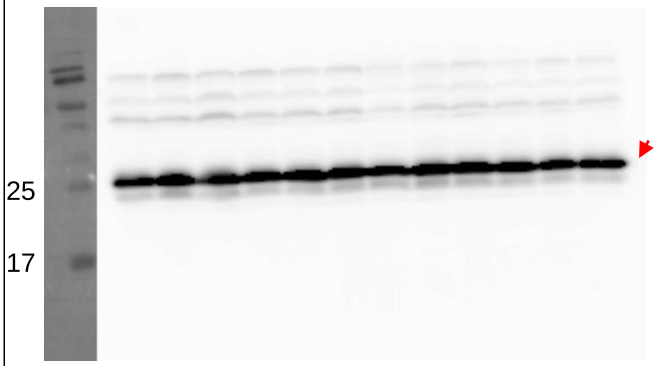

Cas3

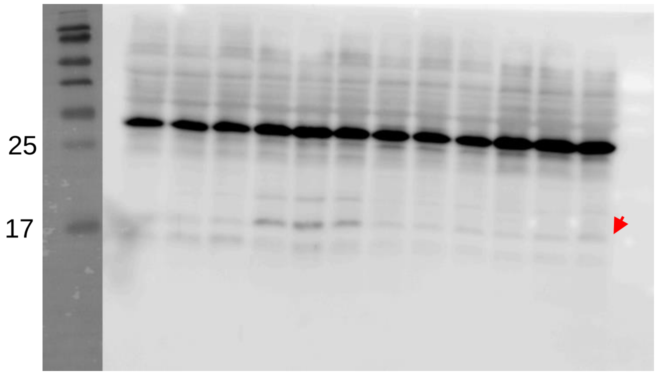

C-Cas7

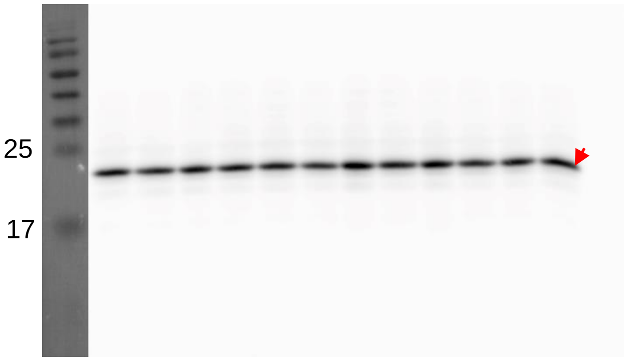

Cas7

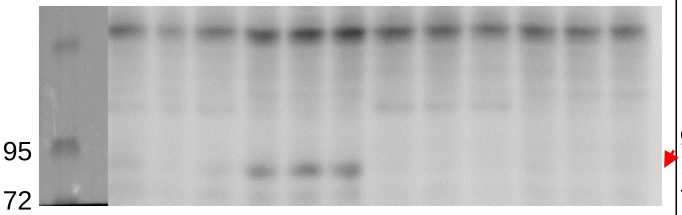

c-PARP

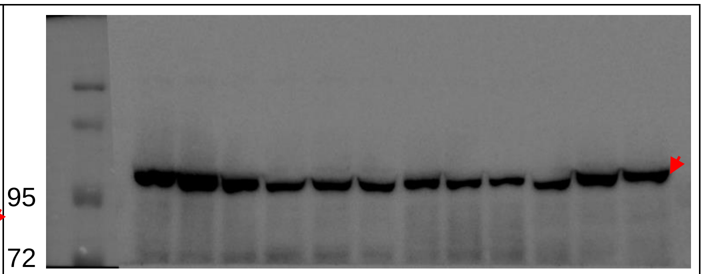

PARP

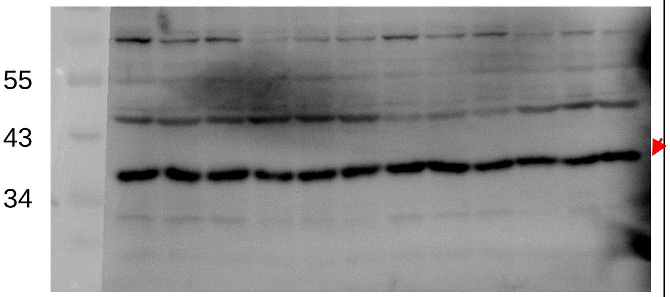

GAPDH
