# Supplementary material for: The matricellular protein CCN5 induces apoptosis in myofibroblasts through SMAD7-mediated inhibition of NFκB
Source: PLoS One. 2022 Aug 2;17(8):e0269735. doi: 10.1371/journal.pone.0269735 (PMC9345366; doi:10.1371/journal.pone.0269735)
Supplement: Attachment — Submitted filename: PONE_comments.docx [file pone.0269735.s004.docx]

The Manuscript "The matricellular protein CCN5 induces apoptosis in myofibroblasts through SMAD7-mediated inhibition of NFkB" by Nguyen et al is the extension of their earlier work where they have shown that the matricellular protein CCN5 reverses established cardiac fibrosis (CF) through inducing apoptosis in myofibroblasts (MyoFBs) but not in cardiomyocytes or fibroblasts (FBs). In this study, authors have the elucidate the molecular mechanisms underlying CCN5-mediated selective apoptosis of MyoFBs. Authors have demonstrated that the role of CCN5 in MyoFB-specific apoptosis using cardiac MyoFBs freshly isolated from rat hearts and trans-differentiated by treatment with TGF-β. They found that a balance between pro-apoptotic p53 and anti-apoptotic NFkB is established in MyoFBs and that CCN5 tips over the balance to favour p53, which results in apoptosis through SMAD7-mediated inhibition of NFkB. In the end, the authors have claimed that this work provides insight into the role of CCN5 in reversing CF and a basis for the development of anti-fibrotic therapies.

Authors have adequately addressed my comments raised in a previous round of review and I feel that this manuscript is now acceptable for publication. One suggestion to author, should mentions the three and four asterisk (*** and ****) in figure legend-3, legend-4 and legend-5.
